# Comparison of the Performance of Five Generative Artificial Intelligence Models on a Medical Molecular Biology Examination

**DOI:** 10.7759/cureus.88480

**Published:** 2025-07-21

**Authors:** Xiaoying Jiang

**Affiliations:** 1 Department of Biochemistry and Molecular Biology, Xi'an Jiaotong University, Xi'an, CHN

**Keywords:** artificial intelligence, basic medical sciences, medical education, medical molecular biology, medical undergraduates

## Abstract

Objective

The aim of this study is to evaluate the performance of five common Chinese generative artificial intelligence (GAI) models on a medical molecular biology examination and assess the application value of these GAIs in teaching.

Methods

A set of medical molecular biology test questions was used to measure the performance of five Chinese GAIs, including ERNIE Bot, chatGLM, iFLYTEK Spark, Qwen, and Doubao. The correct response rates of the five GAIs were compared with those of actual medical undergraduates using an unpaired t-test in GraphPad Prism 6.01.

Results

The total scores of the five GAIs all exceeded the passing score of 60 (full score: 100), ranging from 75.67 to 88.67. ERNIE Bot, chatGLM, Qwen, and Doubao demonstrated higher correct rates for total scores (80.33%-88.67%; p-value: 0.0127-0.0492) and for multiple-choice questions (83.33%-87.50%; p-value: 0.0071-0.0137) compared to actual undergraduates, showing a different distribution pattern of incorrect responses.

Conclusion

This study demonstrated the effectiveness of the five GAIs as learning aids in medical molecular biology. However, due to occasional incorrect answers, undergraduates should apply critical thinking when using GAI-generated responses. Meanwhile, a discipline-specific AI agent for medical molecular biology should be developed as soon as possible.

## Introduction

In recent years, generative artificial intelligence (GAI) has advanced at an unprecedented pace, profoundly impacting human society and revolutionizing daily life, particularly in the field of education [1]. As a milestone in the history of GAI development, ChatGPT, launched by OpenAI in 2022, demonstrates remarkable capabilities: it can understand users' queries and generate a diverse range of outputs within seconds, including natural language texts, images, slide presentations, audio, video, and other digital content [1,2]. Powered by large language models (LLMs) and machine learning algorithms, ChatGPT can not only answer various questions in contextual dialogues resembling human conversations, but also write academic papers, create poetry and music, and develop computer programs. The extensive capabilities of ChatGPT have driven its rapid global adoption, amassing over 100 million active users within two months of release [3,4]. In April 2023, the United Nations Educational, Scientific and Cultural Organization (UNESCO) released a guide titled ChatGPT and Artificial Intelligence in Higher Education: Quick Start Guide, aimed at facilitating the integration of GAI into higher education [5].

Unarguably, GAI will also bring transformative opportunities and challenges to higher medical education [6,7]. As a pedagogical tool, GAI holds significant potential to augment teaching resources, diversify instructional methodologies, and enable adaptive learning systems capable of real-time question-answering, thereby optimizing educators' workflow efficiency and students' self-directed training [6]. Empirical evidence from ChatGPT applications has demonstrated its efficacy in medical education, such as providing personalized learning materials, designing virtual clinical scenarios, and assisting in writing scientific articles [7]. The individual learning style of students affects the final outcomes of learning. Each student, having their own learning style and preferences, plays a significant role in their motivation and learning, with or without AI [8].

Molecular biology is a cornerstone discipline in the medical curriculum. It reveals the molecular pathogenesis of human diseases and is a critically important foundational course for medical undergraduates [9,10]. Although GAI has already been applied in many clinical disciplines, including ophthalmology, plastic surgery, and hepatobiliary surgery, it has not yet been reported in the teaching of medical molecular biology [11-13]. Furthermore, geopolitical constraints limit Chinese learners' access to ChatGPT, necessitating the evaluation of domestically developed GAI platforms. To address this gap, we assessed the performance of five prominent Chinese GAI systems (ERNIE Bot, chatGLM, iFLYTEK Spark, Qwen, and Doubao) on a medical molecular biology examination, aiming to establish their pedagogical applicability for medical undergraduates in China.

## Materials and methods

Study design and ethics

This study was designed and conducted in May 2024 by the author from the Department of Biochemistry and Molecular Biology at Xi'an Jiaotong University Health Science Center. The author has over two decades of teaching experience in medical molecular biology. To evaluate the domain-specific proficiency of free Chinese GAI in medical molecular biology, the author developed a set of test questions aligned with the molecular biology curriculum for five common Chinese GAI interfaces (ERNIE Bot, ChatGLM, iFLYTEK Spark, Qwen, and Doubao). Ethics approval was not applicable given the nature of this study.

Study procedure

The test set contained 40 multiple-choice questions, 6 short-answer questions, and 3 essay-type questions, simulating the actual molecular biology examination for Chinese medical undergraduates in terms of question types, difficulty, and scope. The textbook used at our university was the ninth edition of Biochemistry and Molecular Biology, published by People's Medical Publishing House [9,10]. The author input these examination questions into each GAI platform’s API interface (ERNIE Bot, ChatGLM, iFLYTEK Spark, Qwen, and Doubao) three times. The author collected the answers from these GAIs and determined the correctness of each response.

Analysis of scores

The performance of five GAI answers on this simulated examination of medical molecular biology was compared to the performance of 12 actual Chinese undergraduates. The author taught medical molecular biology to these actual undergraduates and provided the offline examination containing similar questions: 40 multiple-choice questions, 6 short-answer questions, and 3 essay-type questions. The author also determined the correctness of these actual undergraduates’ answers.

Statistical analysis

The data were represented as Mean ± SEM. The correct rates of the five GAIs and actual undergraduates on total scores, multiple-choice questions, short-answer questions, and essay-type questions were analyzed using an unpaired t-test with GraphPad Prism 6.01. A p-value of less than 0.05 was considered to be statistically significant.

## Results

Performance of five free Chinese GAI

**Figure 1:**
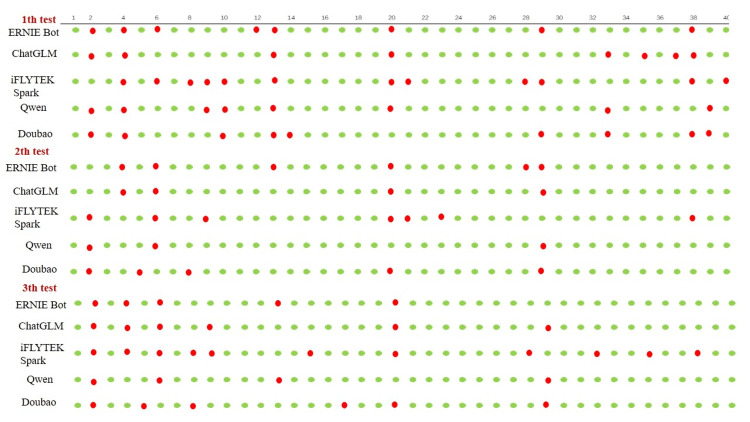
The distribution of wrong answers by GAIs on multiple-choice questions. A green ball indicates a correct answer, and a red ball indicates a wrong answer. GAI: Generative artificial intelligence.

In May 2024, the performance of five free Chinese GAI on the domain-specific knowledge of medical molecular biology was evaluated based on their responses to a set of examination questions. The total scores of the five GAIs were all above the passing score of 60 (total score: 100): ERNIE Bot (85.33 ± 1.667), ChatGLM (80.33 ± 1.453), iFLYTEK Spark (75.67 ± 2.667), Qwen (85.00 ± 0.5774), and Doubao (88.67 ± 1.453) (Table 1). The correct rates of the five GAIs were 75.17%-87.50% on multiple-choice questions, 75.57%-91.10% on short-answer questions, and 75.53%-93.30% on essay-type questions (Table 1). A green ball indicates that the GAI provided a correct answer, while a red ball indicates a wrong answer. All the answers of the five GAIs on multiple-choice questions are shown in Figure 1, suggesting that the number of incorrect answers varied among the five free GAIs. Repetitive questioning did not improve the accuracy of the answers (Figure 1).

**Table 1:** Correct answer rates (%) of five free GAIs on the Medical Molecular Biology examination compared with real undergraduates. GAI: Generative artificial intelligence.

Testers	Multiple-Choice Questions		Short-Answer Questions		Essay-Type Questions		Total Score	
	Mean ± SEM	p-value	Mean ± SEM	p-value	Mean ± SEM	p-value	Mean ± SEM	p-value
ERNIE Bot	84.17 ± 2.205	0.0118	82.23 ± 2.936	0.0611	90.00 ± 0.00	0.0468	85.33 ± 1.667	0.022
ChatGLM	85.00 ± 2.887	0.0104	75.57 ± 1.133	0.1423	78.90 ± 1.100	0.2028	80.33 ± 1.453	0.0492
iFLYTEK Spark	75.17 ± 3.768	0.0507	76.67 ± 5.106	0.1287	75.53 ± 2.233	0.2997	75.67 ± 2.667	0.1021
Qwen	87.50 ± 3.819	0.0071	77.77 ± 4.467	0.1112	88.87 ± 2.942	0.0559	85.00 ± 0.5774	0.023
Doubao	83.33 ± 3.005	0.0137	91.10 ± 1.100	0.0176	93.30 ± 0.00	0.0292	88.67 ± 1.453	0.0127
Real students	49.58 ± 5.723	–	54.58 ± 6.527	–	61.46 ± 6.318	–	55.25 ± 5.614	–

Comparison of the performance of five GAI and real undergraduates

The performance of the five Chinese GAIs on the examination of medical molecular biology was then compared to that of actual undergraduates using the same types of examination questions, with the same difficulty level and scope. The author designed and graded the examination questions for both the GAIs and the actual undergraduates. Compared with the undergraduates, ERNIE Bot, ChatGLM, Qwen, and Doubao showed statistically higher correct answer rates on the total score and on multiple-choice questions. ERNIE Bot and Doubao also showed statistically higher correct rates on essay-type questions compared with the actual undergraduates. Only Doubao showed a statistically higher correct rate on short-answer questions compared with the undergraduates (Table 1).

## Discussion

Emerging evidence demonstrates that AI will greatly transform the pattern of education and reshape the process of teaching and learning for both teachers and students [14-16]. Along with the accelerated evolution of AI, educators should cultivate an adaptive learning mindset by systematically acquiring AI literacy and strategically implementing AI-integrated pedagogical frameworks. Currently, AI-driven training systems are becoming increasingly important in medical education, such as robot-assisted surgical systems and AI imaging systems [17-19]. With the growing use of AI in medical education, medical undergraduates may increasingly rely on AI to complete their academic assignments or seek answers to professional questions [20,21]. Therefore, medical educators should guide students on how to critically engage with AI-generated content.

ChatGPT has been evaluated for its performance on the Physician Licensing Exam in Taiwan, the Japanese Medical Licensing Exam, and the U.S. prosthodontic exam [22-24]. The performance of GPT-4o and DeepSeek-R1 has also been compared on the Polish specialty examination in infectious diseases [25]. Since ChatGPT is not freely available to users in China, we assessed the performance of free Chinese GAI (ERNIE Bot, ChatGLM, iFLYTEK Spark, Qwen, and Doubao) on the medical molecular biology examination, as these are commonly used by Chinese students.

When medical undergraduates ask these general GAI for academic problems, undergraduates are not sure whether the answers from GAI are totally correct or not. Therefore, it is very necessary to know the accuracy of the answers from these GAI used by medical undergraduates. In this study, five free GAI all obtained total scores beyond the routine passing score of 60, ranging from 75.67 to 88.67, but not all answers were correct across the five GAI. Similarly, ChatGPT-3.5 achieved an average accuracy of 67.7%, while ChatGPT-4 showed average accuracies of 91.9% in basic medical sciences of the Taiwan physician licensing exam [22]. GPT-4V scored 85.1% on the essential knowledge section of the Japanese medical licensing exam [23]. GPT-4o correctly answered 85 out of 199 questions, while DeepSeek-R1 correctly answered 88 out of 199 questions in the Polish infectious diseases specialty exam [25]. In special medical fields, these general AI did not provide 100% accurate answers. Therefore, it is very necessary to develop a discipline-specific AI agent for medical molecular biology for students.

Study limitations

The current study has several limitations. First, these general GAI used to test the response to medical molecular biology did not cover all Chinese GAI that can be freely used now. Second, we have not established the domain-specific AI agent of medical molecular biology. Although GAI is evolving very rapidly, many problems in the medical field require specific expertise and contexts, making it challenging for these general GAI to provide correct answers. It is essential that medical educators and students understand the limitations of AI tools. These general GAI need to be trained with more professional knowledge of medical molecular biology. We believe that a deeper understanding of GAI is the basis to better apply GAI for teaching.

## Conclusions

These five free Chinese GAIs (ERNIE Bot, ChatGLM, iFLYTEK Spark, Qwen, and Doubao) all achieved total scores ranging from 75.67 to 88.67 (full score: 100) on the medical molecular biology examination, confirming that these GAIs could serve as an assistance tool for medical undergraduates. However, the incorrect answers from AI highlight the importance of developing a discipline-specific AI agent as soon as possible.
